# Supplementary material for: Nemo: a computational tool for analyzing nematode locomotion
Source: BMC Neurosci. 2007 Oct 17;8:86. doi: 10.1186/1471-2202-8-86 (PMC2148042; doi:10.1186/1471-2202-8-86)
Supplement: Additional file 1 — Steps ensued for quantitative analysis. Description of the steps ensued for quantitative analysis and features of the algorithm and the GUI. [file 1471-2202-8-86-S1.pdf]

Nemo: A computational tool for analyzing nematode locomotion

George D. Tsibidis[1§] and Nektarios Tavernarakis[2]

[1]Institute of Electronic Structure and Laser, Foundation for Research and Technology, P.O.Box 1385, Vassilika Vouton, 71110 Heraklion, Crete, GREECE

[2]Institute of Molecular Biology and Biotechnology, Foundation for Research and Technology, P.O.Box 1385, Vassilika Vouton, 71110 Heraklion, Crete, GREECE

Description of the steps ensued for quantitative analysis and features of the algorithm and the GUI

1. Image analysis and object extraction

The first step in image analysis is the removal of noise usually introduced during the acquisition of images. A mean low-pas filter is applied to smooth images (Fig.1A), which replaces every pixel with the average of its 3x3 neighbourhood. The freeware ImageJ software is used to perform the filtering operation on all images in a fast way. Every image is subsequently segmented by subdividing it into its constituent objects. Segmentation is performed by means of a threshold value *T* defined by the user and as a result, binary images are produced (Fig.1B). Black pixels correspond to background while white pixels correspond to the worm.

In order to extract useful quantitative information about the worms in the frame, a number of morphological operations are applied on the binary images. Dilation is one of the basic operators used to gradually enlarge the boundaries of regions of foreground pixels. Thus, areas of foreground pixels grow in size while holes within those regions become smaller. This is a very important morphological operation because it helps to clean small spots inside the animal body. Connected components labelling scans every image and groups its pixel into components based on pixel connectivity. Large objects are assigned to worms in the image while smaller objects outside the perimeter of the animals are removed. Using built-in functions provided by the Image Analysis Toolbox of Matlab, the perimeter of each worm can be easily obtained (Fig.1C). The 'spine' (or 'skeleton') of the animal is obtained in the same fashion (Fig.2). An algorithm has been also developed to remove small 'branches' on the skeleton (Fig.3). The skeleton correction is achieved by removing redundant branches of the skeleton manually. The image processing procedure is summarised in Fig.4.

The distinction of the head from the tail of the animal is an issue that is necessary to be addressed properly. In principle, the head of the worm is less dark than the tail, a feature constituting the basis for distinguishing the two parts of the body. The procedure, however, we follow to determine the front and back part is accomplished by minimum intervention by the user: the user decides in the first frame of the sequence the head and the tail (point A and B in Fig. 5A, respectively), in an interactive way. In the second frame, the skeleton has again two endpoints A' and B' (Fig.5). We assign '*Head*' to be the endpoint corresponding to *min(distance(A,A'),*

distance(A,B')). Similarly, *'Tail'* is determined by *min(distance(B,A'), distance(B,B'))*. The same procedure is repeated for the rest of the images in the video sequence comparing distances in successive frames. This approach is correct provided that the distance between the head and the tail is large enough that the animal does not form a closed loop.

Development of the algorithm

The algorithm we developed aims to produce a number of parameters that can be used to analyse the movement and behavioural phenotype of the worm. After the initial extraction of the area occupied by the animal, the attention is restricted only to small boxes containing the animal (Fig.6). The advantage of reducing the region of interest analysis is that it allows to avoid time consuming computations.

The total number of white pixels representing the mask in the binary counterpart of the raw image yields the area of the animal in pixels. Given the length of the *C.elegans*, which is approximately 1mm, the area occupied by the animal in physical units can be computed. By means of built-in functions from the Matlab Image Analysis Toolbox, the perimeter points of all nematodes in the video sequence are determined.

The skeleton of the animal helps to compute the length of the animal adding the sum of the distances between adjacent pixels along the spine of the animal. Knowing the approximate equivalence of the physical units to pixel size, the actual size of the worm spine can be calculated.

In order to analyse the movement of the animal, the coordinates of points along the spine must be computed. To this end, the body of the worm is divided into a number of segments *N* (in this experiment *N*=7 as seen in Fig.7). The position of the centre of mass of every part (Fig.8A) as well as the centroid of the whole animal are recorded (Fig.8B). The centre of mass of every segment is approximated by computing the centre of mass of the skeleton section in this segment. A more precise but time consuming procedure would require the calculation of the coordinates of the centre of mass considering all body pixels within that particular segment. It emerges, though, the correction in the estimation of the centre of mass is small compared to the time required to complete the calculations. It should be emphasised that in computing the centre of mass of the entire animal, the front part (first segment) has been ignored. In principle, the head of the worm possesses more degrees of freedom and it moves more abruptly than the rest of the body. The inclusion of the front part of the animal in the calculations could then lead to a big fluctuation to the position of the body centroid.

It is also possible that the worm might move outside the view of the camera during the video sequence and therefore it is necessary to use an absolute reference coordinate frame. In other studies, an automated imaging system has been built that follows the objects based on the threshold difference between the animals and the background. By contrast, our algorithm takes into account stationary points in the images as reference points for reducing the coordinate value according to an absolute reference frame.

The thickness of the animal is also computed in all the segments except from the front (head) and back (tail) because the end parts of the animal are thinner. In order to compute the thickness of each segment, a minimum intervention by the user is required. More specifically, we use the binary image of the worm with the perimeter, skeleton and centre of mass of the segment sketched in the picture (Fig.9A). We define a quantity *W* to be equal to half of the thickness of the segment. The user chooses a circle of a radius bigger than *W* in such a way that it will ensure intersection with the perimeter in four points. These intersections define two segments along the perimeter and the distance between each point and the centre of mass is computed. The average of the smallest distances determines approximately *W* (Fig.9B) and thereby the thickness can be easily estimated. The user is required to intervene only in the first frame to define the circle radius. This value is passed to the rest of the frames and the automated procedure is very fast and efficient.

Management of data and calculation of other parameters

A Graphical User Interface (Fig.10) has been developed aiming to provide the means to assist the researcher in analysing the collected data in a fast and efficient way. It operates as an information management tool and it allows the user to conduct further calculations. Graphs and histograms of measurable and computed parameters can be plotted and image figures can be produced by the GUI:

(a). Images:

Image figures can show raw frames with the worm, its perimeter, spine, segments, centre of mass of the entire animal, centre of mass of individual segments. As a result, the GUI offers an interactive tool to see all the above morphological quantities at any

time point. The total trajectory of the centre of mass (*CM_segment*) of any segment of the worm skeleton can be also obtained and illustrated (Fig.11) and subsequently analysed. X-coordinates and Y-coordinates of *CM_segment* as a function of time can be plotted (Fig.12).

(b). <u>Computation of other parameters:</u>

The knowledge of the location of particular points (centre of mass of the animal or centroids of segments) can facilitate the calculation of other parameters. In principle, the size of the animal is big enough to lead to a good and accurate estimation of the position of these points. It is, however, possible even a small deviation to provide meaningless patterns. For example, when the angle in the frame i,

$$\phi_i = \arctan\left(\frac{y_{i+1} - y_i}{x_{i+1} - x_i}\right)$$

is calculated, where y and x are the position coordinates of the point in successive frames, the results demonstrate big fluctuations (Fig.13A). To eliminate this behaviour, position coordinates are averaged over a number of frames helping to obtain smoother results (Fig.13B). This assumption is reasonable if the animal does not move very fast. The GUI allows the researcher to choose the number of frames over which data will be averaged (i.e. in this experiment, the default value is 4).

1. *Distance covered by the worm*

The distance covered by the worm is defined as the length of the total trajectory of the centre of mass (CM) of the animal. The distance between CM in successive frames is computed and then the sum represents the total length of the trajectory. In the current experiment, the distance covered equals 5.55mm.

2. *Thickness of the animal*

The average thickness of the animal as derived by considering all values produced by the automated algorithm (i.e. 0.092mm). In Fig.14A, the histogram of the values is plotted.

3. *Distance between head and tail of the worm*

In principle, the worm moves on a sinusoidal line, and thereby, the distance between the end-points will not equal the length of the animal. In Fig.14B, the histogram of values of this distance is sketched.

4. *Speed of CM or CM_segment*

The displacement of the position of a point over time yields the speed of *CM* and *CM_segment* and the plots are illustrated in the form of a time series or a histogram in Fig.15A and Fig.15B, respectively.

5. *Wavelength*

The calculation of the wavelength of the sinusoidal movement of the C.elegans is conducted by means of observing the change in the angle ϕ_i (Fig.13B). The user uses a region where the pattern appears to be reasonably smooth where there exists a clear wave-like behaviour. The positions between two successive maxima (or minima) can be used to define the wavelength of the sinusoidal movement. In Fig.16A, the movement of the centre of mass of the second segment of the animal is illustrated. The line between two successive asterisks represents a wavelength (i.e. 0.37mm).

6. *Amplitude*

By applying a transform on the coordinates of the trajectory of the centroid of a particular segment, the waveform amplitude can be obtained. In Fig.16B, the sinusoidal waveform of the movement of the second segment is plotted yielding an approximate value for the amplitude equal to 0.03mm.

7. *Turning*

Various reasons can result in a deviation from a smooth sinusoidal movement of the animal (Fig.11). In principle, these reasons are related to morphological changes in the neighbourhood or other behavioural processes. To quantify the directional change, we have computed the angle change between successive wavelengths (Fig.17).

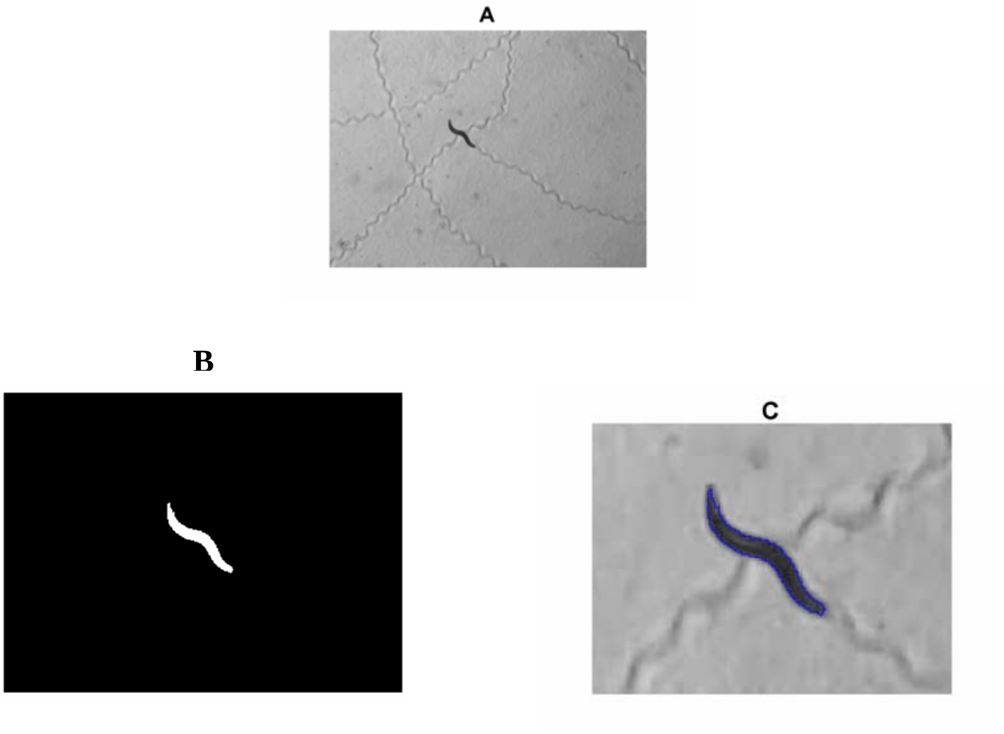

Figure 1 - Image processing of the image

(A) Raw Image. (B) Mask after thresholding and morphological operations. (C) Perimeter of the worm.

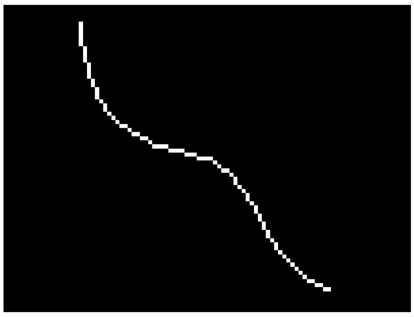

Figure 2 - Skeletonisation of the image I

(A) skeleton with branches. (B) skeleton after the application of the algorithm to remove the branches.

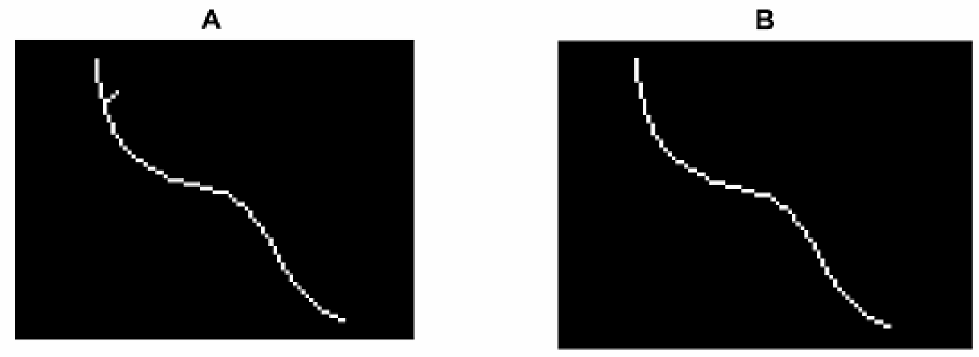

Figure 3 - Skeletonisation of the image II

(A) skeleton with branches. (B) skeleton after the application of the algorithm to remove the branches.

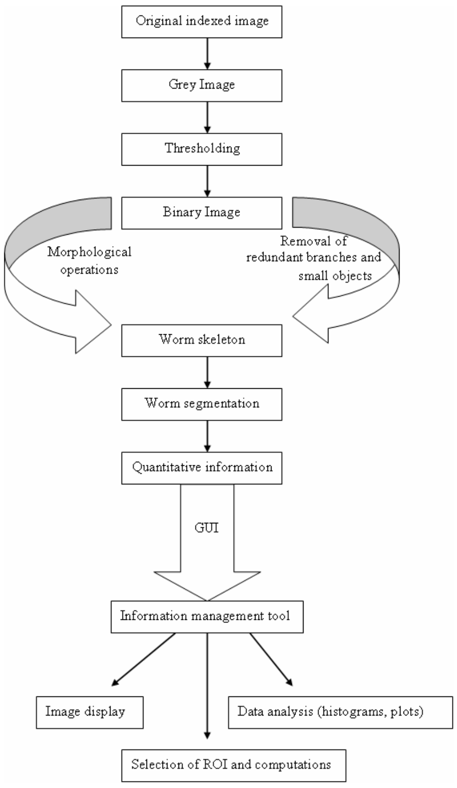

Figure 4 - Image analysis procedure

Steps followed during the image analysis procedure.

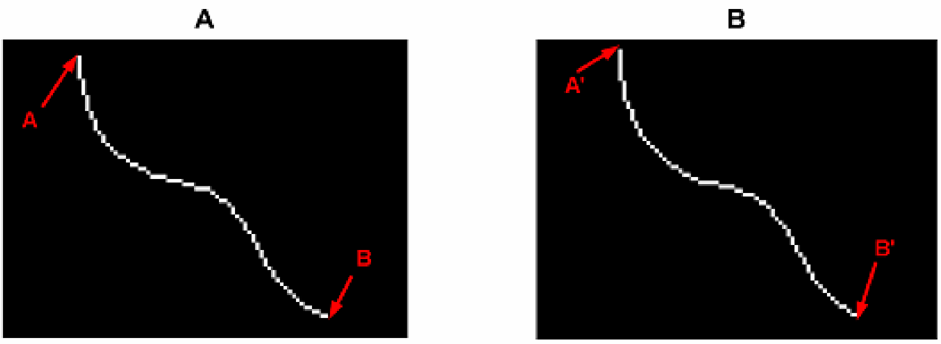

Figure 5 - Head and tail of the worm in two successive images

(A) A and B represent the head and tail of the animal. (B) A' and B' are the endpoints of the skeleton and the minimisation of the distances will determine whether they can be assigned to the 'head' or 'tail'.

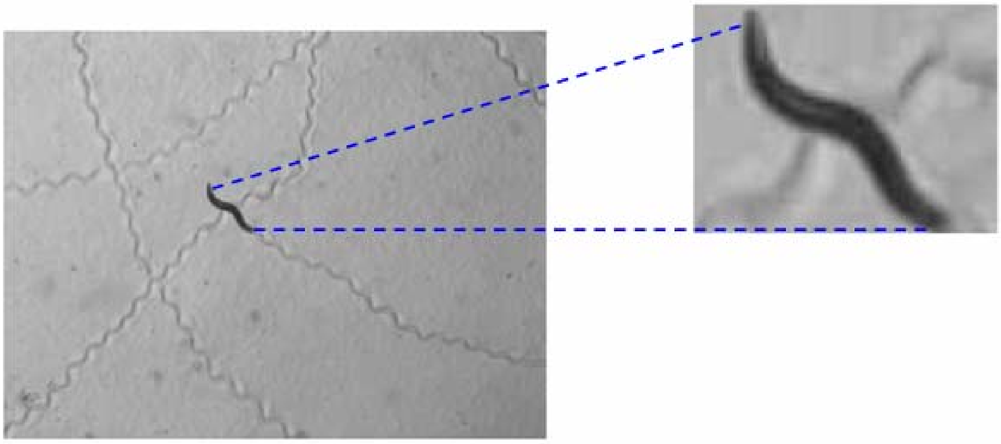

Figure 6 - Reduction of area studied

An image analysis on a smaller area (box bounding animal) increases calculation speed.

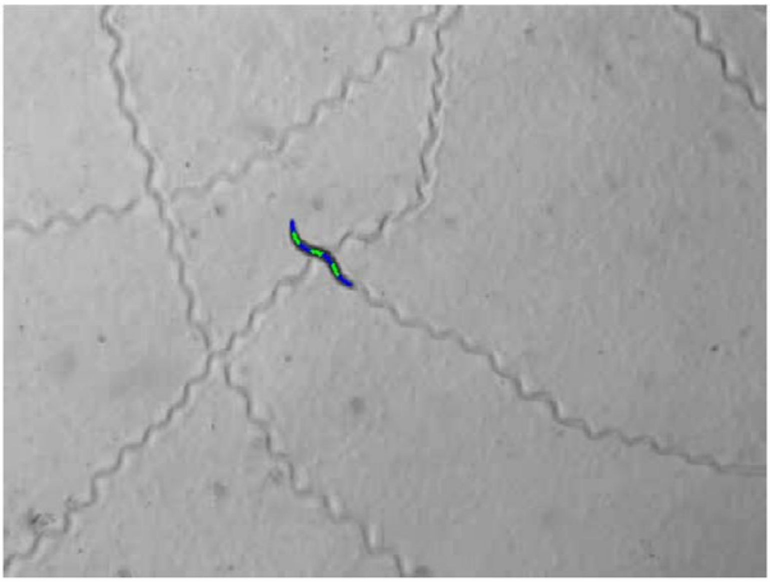

Figure 7 - Worm segmentation

Worm segmentation (only the skeleton is depicted).

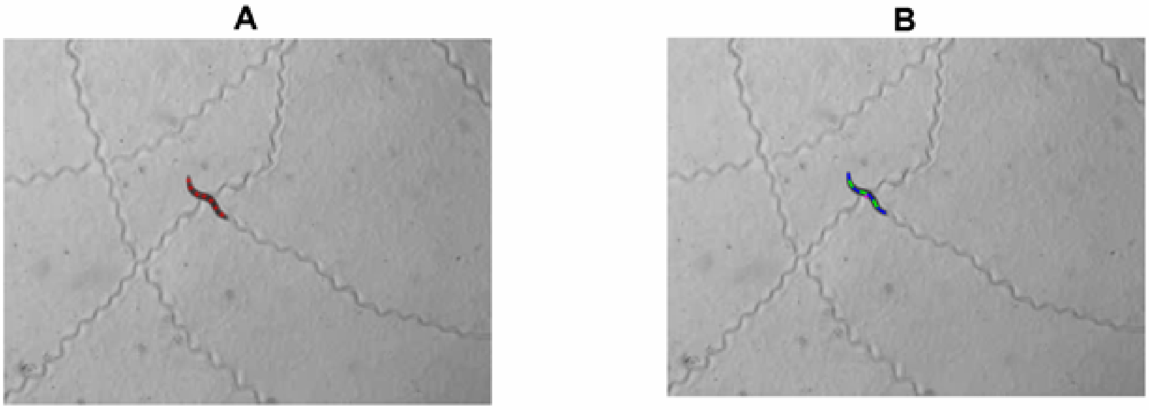

Figure 8 - Spine segments and centroid of animal

(A) The skeleton of the animal and the Centroid of each segment (red stars). (B) The skeleton of the animal and the centre of mass of the entire worm (magenda star).

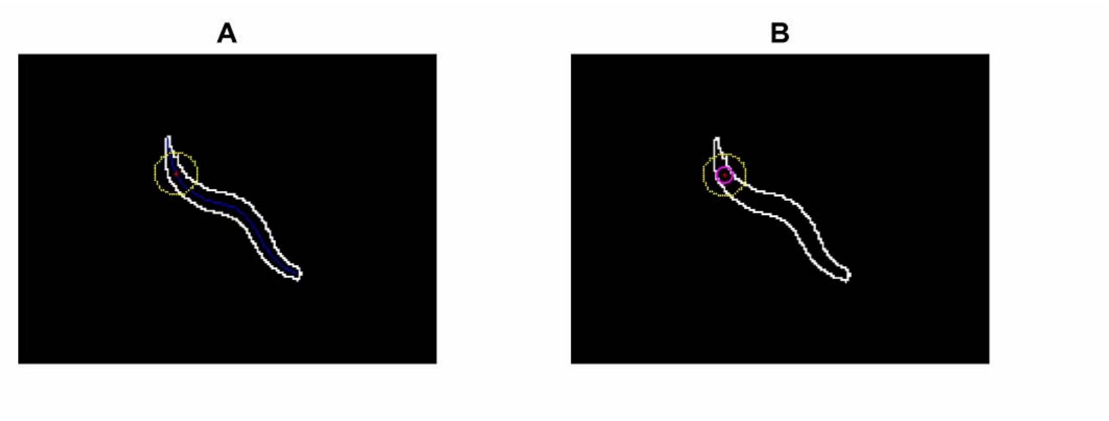

Figure 9 - Thickness of the animal

(A) perimeter of the animal and centroid of the first segment (red star). Yellow line represents the circle chosen by the user. (B) Inner circle has a size that equals half the thickness of the segment.

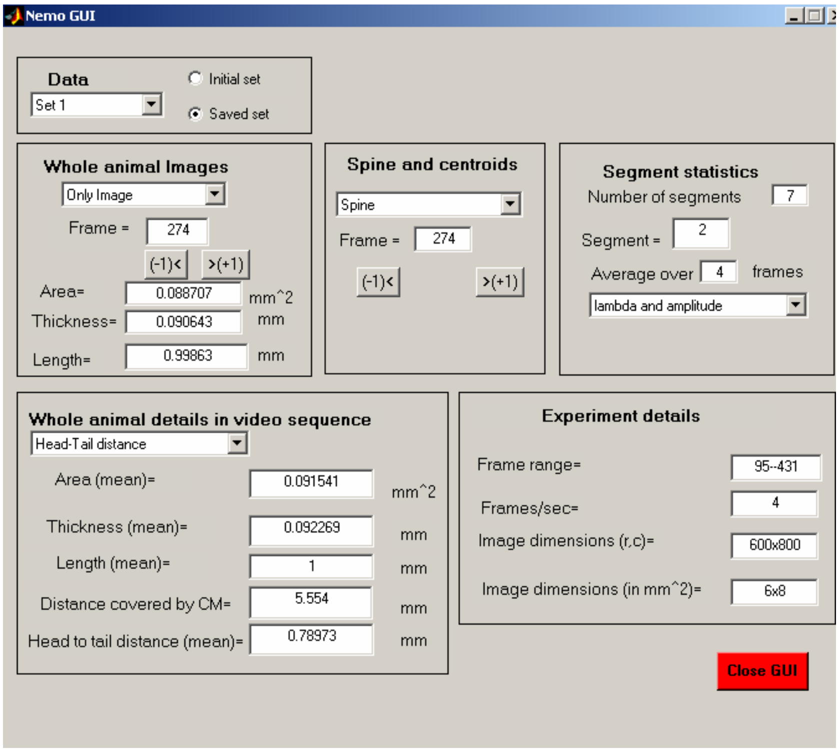

Figure 10 - Graphical User Interface

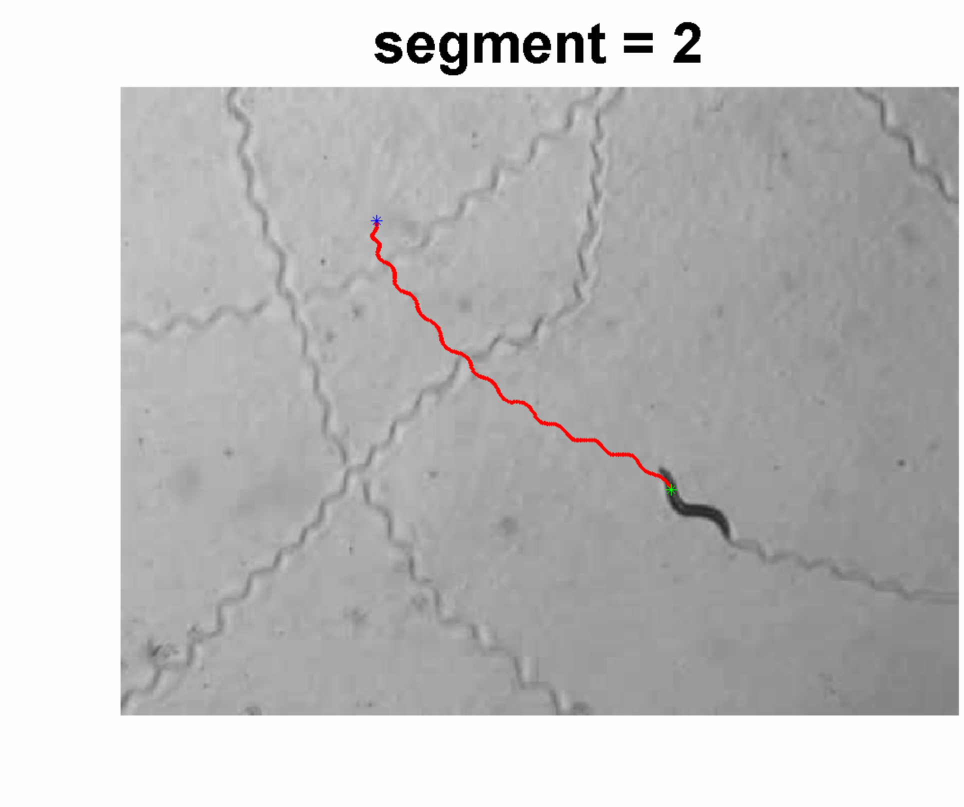

Figure 11 - Trajectory of the centre of mass of a segment

Trajectory of centre of mass of segment 2 of the worm (green star: starting point, blue star: final point)

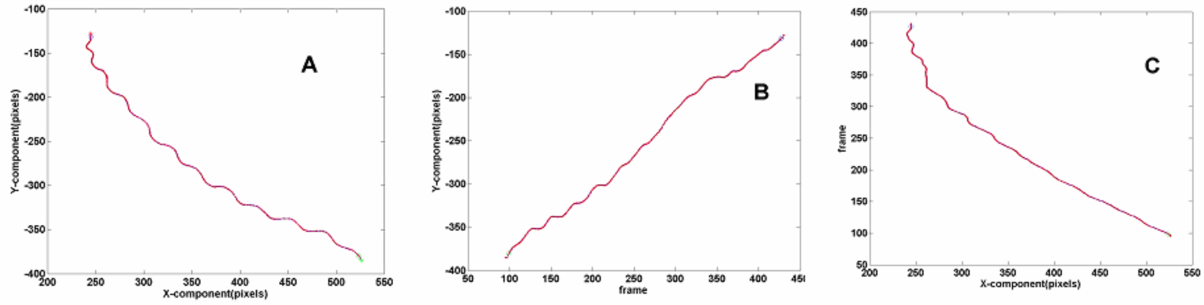

Figure 12 - Graphical User Interface functions

(A) trajectory of centre of mass (*CM_segment*) of segment 2. (B) Y-coordinate of *CM_segment* . (C) X-coordinate of *CM_segment* . (green star: starting point, blue star: final point).

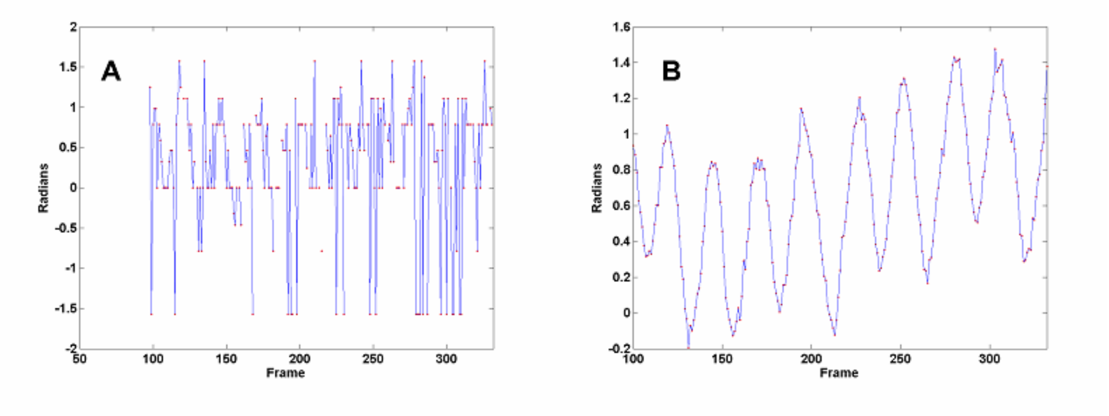

Figure 13 - Angle change

(A) Angle evolution with respect to time before averaging. (B) Angle evolution with respect to time after averaging.

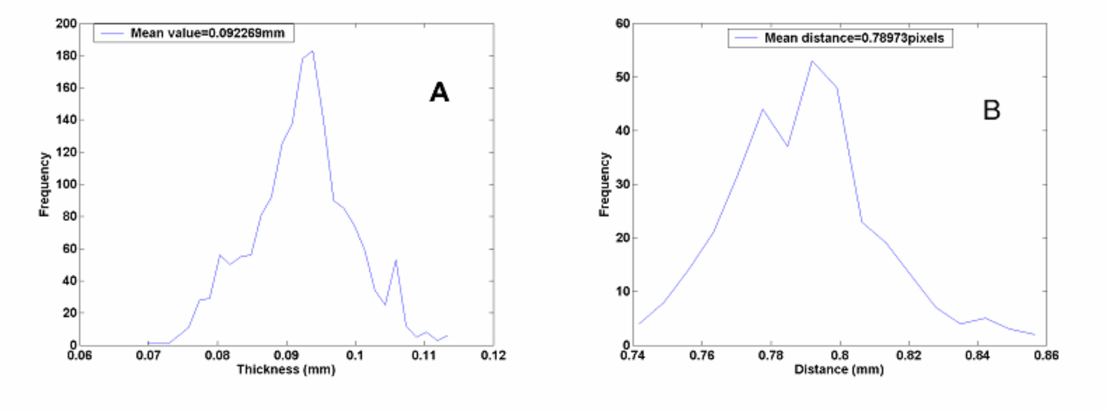

Figure 14 - Thickness and distance between head and tail

(A) Thickness histogram. (B) Distance between head and tail.

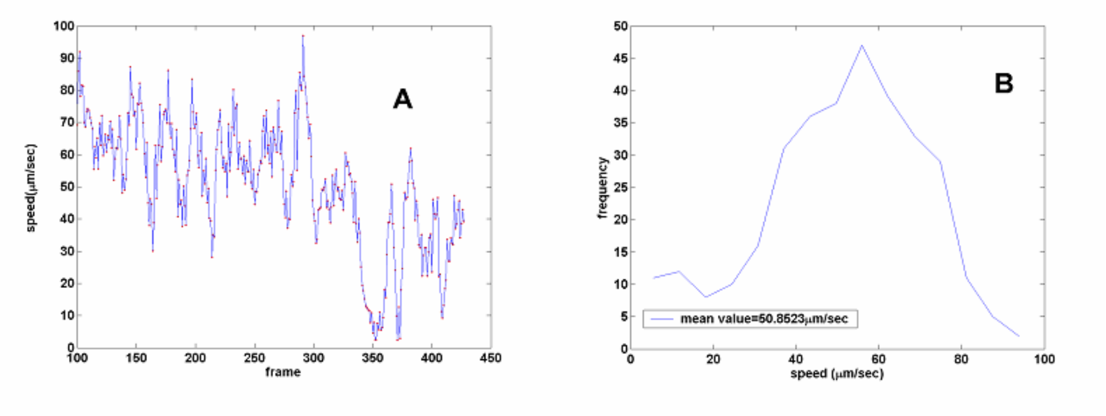

Figure 15- Speed

(A) Speed series for segment 2. (B) Speed histogram for segment 2.

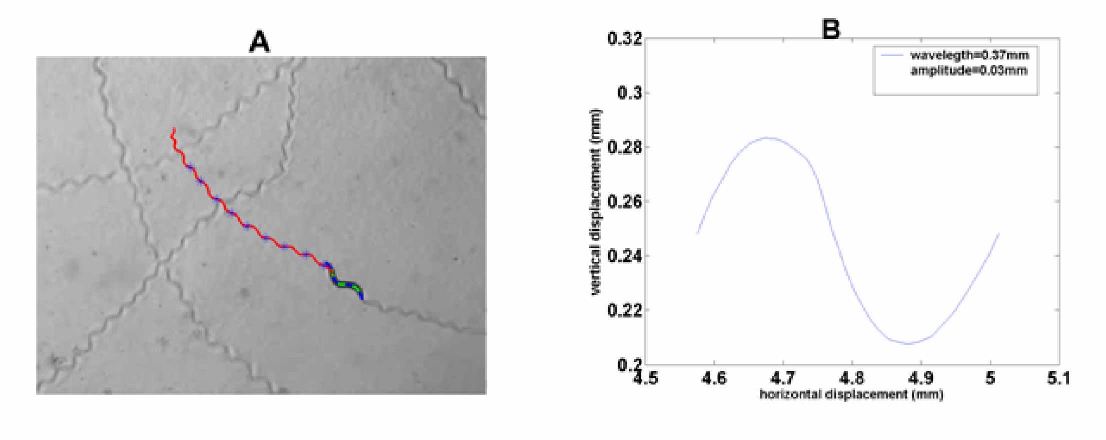

Figure 16 - Features of sinusoidal movement

(A) Trajectory of centre of mass of segment 2. The length between two successive blue asterisks equals one wavelength. (B) Wavelength and amplitude of sinusoidal movement.

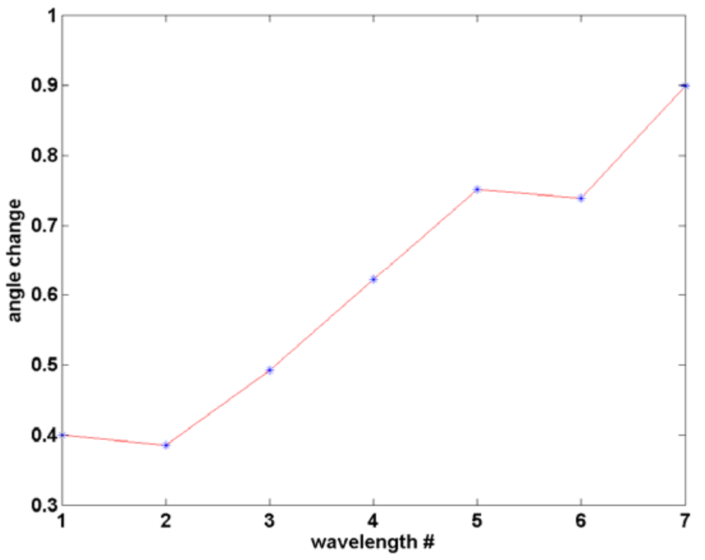

Figure 17 - Angle change

Total angle change in every wavelength.

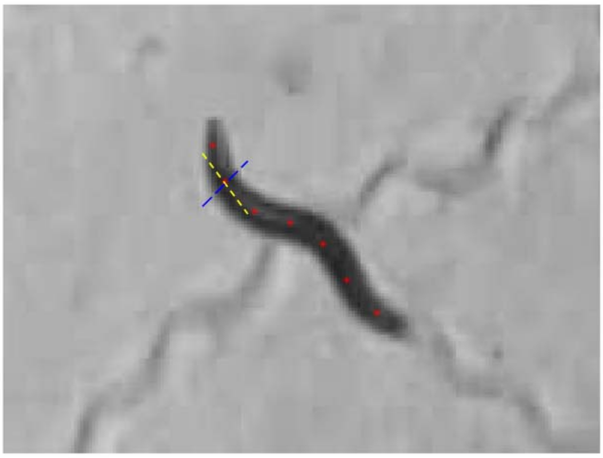

Figure 18 - Thickness computation

A different approach for thickness computation: Yellow line goes through the centre of mass of the segment while blue line is perpendicular to it.
