# Supplementary material for: Nemo: a computational tool for analyzing nematode locomotion
Source: BMC Neurosci. 2007 Oct 17;8:86. doi: 10.1186/1471-2202-8-86 (PMC2148042; doi:10.1186/1471-2202-8-86)
Supplement: Additional file 2 — Computer algorithms. Codes used to extract quantitative information. [file 1471-2202-8-86-S2.zip › Computer algorithms/Algorithms.pdf]

Nemo: A computational tool for analyzing nematode locomotion

George D.Tsibidis[1§] and Nektarios Tavernarakis[2]

[1]Institute of Electronic Structure and Laser, Foundation for Research and Technology, P.O.Box 1385, Vassilika Vouton, 71110 Heraklion, Crete, GREECE

[2]Institute of Molecular Biology and Biotechnology, Foundation for Research and Technology, P.O.Box 1385, Vassilika Vouton, 71110 Heraklion, Crete, GREECE

File list and Algorithms

1. Algorithms used to extract image and information data

a. extract_all_objects.m: This algorithm incorporates the rest of the algorithms in this section leading to an automated extraction of image and information data. It requires a minimum intervention from the user which is restricted to the introduction of some input values and the distinction between the ‘head’ and ‘tail’ of the worm.

b. extract_object.m: It applies morphological operations to the animals and after thresholding and removal of the smallest objects in the image, the mask and the skeleton of the animal are derived as binary images.

c. correct_skeleton.m: Given the coordinates of the worm in the previous image, the algorithm determines which of the two endpoints of the animal can be associated with ‘head’ and ‘tail’.
d. point_segment.m: Segmentation procedure of the animal is carried out according to the number of segments defined by the user.
e. area_calculation.m: The area occupied by the *C.elegans* is calculated (in pixels).
f. length_elegans.m: The actual length (in pixels) of the worm considering neighbouring points along the spine (‘skeleton’) of the *C.elegans* is computed.
g. thickness_automatic.m: The thickness particular sections of the animal are calculated by a minimum intervention of the user. Thickness is given for every segment in pixels.
h. cm_total.m: The centroid of the animal is calculated.
i. manual_correction: If the worm’s skeleton contains branches, they are removed by using this algorithm.
j. extract_all_objects_amc.m: After running the previous algorithm, an automated procedure can be used to obtain the revised information related to the skeleton, length, etc (see previous steps) of the worm.
k. Wormloc.m: the file that contains the commands to perform a specific action when the Graphical User Interface is used.
l. Wormloc.fig: the file that needs to run to load the GUI.

2. Data files produced by the algorithms

a. thickness_image=A_segment=B.txt: text file containing a number that represents half the width (in pixels) of segment *B* in image *A*.
b. skeleton_A: binary image that contains the skeleton of the aninal.
c. skeleton_A_point=B: text file that contains the coordinates of the cetroid of the segment *B* in image *A*.
d. skeleton_A_total_point=B: the location of the centre of mass of the animal in image *A* when the animal has been segmented in *B* parts.
e. mask_a: binary image of the animal’s mask.
f. number_of_time_points.txt: text file that contains the number of segments in which the animal has been parted.
g. length_A.txt: actual length of animal’s skeleton (in pixels) considering the spine.
h. frames_per_sec.txt: how many images were taken in the unit of time.
i. edge_A.txt: matrix with the location of the endpoints of the skeleton in images *A* and *A*+1.
j. background_row_column.txt: a text file containing three number corresponding to the threshold (in order to get the binary image) and size of every image.
