# Supplementary material for: Nemo: a computational tool for analyzing nematode locomotion
Source: BMC Neurosci. 2007 Oct 17;8:86. doi: 10.1186/1471-2202-8-86 (PMC2148042; doi:10.1186/1471-2202-8-86)
Supplement: Additional file 2 — Computer algorithms. Codes used to extract quantitative information. [file 1471-2202-8-86-S2.zip › Computer algorithms/readme.pdf]

Nemo: A computational tool for analyzing nematode locomotion

George D.Tsibidis[1§] and Nektarios Tavernarakis[2]

[1]Institute of Electronic Structure and Laser, Foundation for Research and Technology, P.O.Box 1385, Vassilika Vouton, 71110 Heraklion, Crete, GREECE

[2]Institute of Molecular Biology and Biotechnology, Foundation for Research and Technology, P.O.Box 1385, Vassilika Vouton, 71110 Heraklion, Crete, GREECE

README

This directory contains all algorithms and subroutines that make up *Nemo*. The file named *extract_all_objects* is the Matlab file that incorporates all commands of our system. The Graphical User Interface is loaded by typing ‘nemo’ at the Matlab prompt. All files contained here should be placed in the same directory. In order to run the routines, the correct path should be defined in the nemo.m file (**change paths in lines 53 and 185 of the file**).
